# mTOR-inhibitors and post-transplant diabetes mellitus: a link still debated in kidney transplantation

**DOI:** 10.3389/fmed.2023.1168967

**Published:** 2023-05-12

**Authors:** Simona Granata, Silvia Mercuri, Dario Troise, Loreto Gesualdo, Giovanni Stallone, Gianluigi Zaza

**Affiliations:** ^1^Nephrology, Dialysis and Transplantation Unit, Department of Medical and Surgical Sciences, University of Foggia, Foggia, Italy; ^2^Renal, Dialysis and Transplantation Unit, Department of Precision and Regenerative Medicine and Ionian Area (DIMEPRE-J), University of Bari, Bari, Italy

**Keywords:** post-transplant diabetes mellitus, kidney transplantation, immunosuppressive drugs, allograft co-morbidities, mTOR-inhibitor

## Abstract

The mammalian target of rapamycin inhibitors (mTOR-Is, Sirolimus, and Everolimus) are immunosuppressive drugs widely employed in kidney transplantation. Their main mechanism of action includes the inhibition of a serine/threonine kinase with a pivotal role in cellular metabolism and in various eukaryotic biological functions (including proteins and lipids synthesis, autophagy, cell survival, cytoskeleton organization, lipogenesis, and gluconeogenesis). Moreover, as well described, the inhibition of the mTOR pathway may also contribute to the development of the post-transplant diabetes mellitus (PTDM), a major clinical complication that may dramatically impact allograft survival (by accelerating the development of the chronic allograft damage) and increase the risk of severe systemic comorbidities. Several factors may contribute to this condition, but the reduction of the beta-cell mass, the impairment of the insulin secretion and resistance, and the induction of glucose intolerance may play a pivotal role. However, although the results of several *in vitro* and in animal models, the real impact of mTOR-Is on PTDM is still debated and the entire biological machinery is poorly recognized. Therefore, to better elucidate the impact of the mTOR-Is on the risk of PTDM in kidney transplant recipients and to potentially uncover future research topics (particularly for the clinical translational research), we decided to review the available literature evidence regarding this important clinical association. In our opinion, based on the published reports, we cannot draw any conclusion and PTDM remains a challenge. However, also in this case, the administration of the lowest possible dose of mTOR-I should also be recommended.

## Introduction

New-onset diabetes after transplantation (NODAT) refers to the occurrence of diabetes in previously non-diabetic clinically stable kidney transplant recipients who had been discharged from the hospital and tapered to their maintenance immunosuppressive therapy (1).

It occurs in almost 4% to 27% of kidney transplant recipients (2–6) and it may induce the development of the chronic allograft damage by activating several pro-fibrotic mediators [including transforming growth factor beta (TGF-β)] and promoting mesangial matrix expansion and cell hyperplasia (7), accelerating the onset of severe clinical complications/comorbidities.

To diagnose NODAT, the International Consensus Guidelines published in 2003 recommended to use the same diagnostic criteria adopted by the American Diabetes Association and the World Health Organization (ADA/WHO) for type 2 diabetes in non-transplant patients: fasting glucose ≥126 mg/dL (7 mmol/L) on more than one occasion, random glucose ≥ 200 mg/dL (11.1 mmol/L) with symptoms, two-hour glucose after 75 g oral glucose tolerance test of ≥200 mg/dL (11.1 mmol/L), and hemoglobin A1C (HbA1c) ≥ 6.5% (3).

However, since the difficulty to screen all patients before transplantation and to identify pre-existing forms of diabetes, in 2013, a second international consensus changed the nomenclature from NODAT to post-transplantation diabetes mellitus (PTDM; a more inclusive term for diabetes that is diagnosed after transplantation without specific reference to any previous history of diabetes) (3).

Risk factors for PTDM are similar to those for type 2 diabetes mellitus: increased age (>40 years), family history of type 2 diabetes, ethnicity (African-American, Asian, and Hispanic patients are at higher risk compared to Caucasian), abnormal glucose tolerance (expressed by fasting blood sugar levels between 90 and 100 mg/dL), and specific genetic factors (8–12).

Other risk factors for PTDM include the metabolic syndrome and the obesity, responsible for the insulin resistance and the glucose intolerance. Indeed, the adipose tissue from obese subjects increases the expression of Tumor Necrosis Factor-α (TNF-α) (13), which downregulates genes involved in the insulin action (such as glucose transporter (GLUT)-4, insulin receptor, and insulin receptor substrate (IRS)-1) (14, 15), reduces adiponectin release and stimulates adipocyte lipolysis (16).

Also, viral infections may increase the risk of PTDM. Hepatitis C virus (HCV) (17) infection may trigger an immune-mediated reaction against β cells with consequent cytopathic effects, glucose uptake reduction (18), and gluconeogenesis augmentation (19). Likewise, cytomegalovirus (CMV) may directly damage beta-cells by the upregulation of large pro-apoptotic machinery or, indirectly, by facilitating the release of pro-inflammatory cytokines (20).

However, in kidney transplant recipients, the administration of immunosuppressive drugs may further impact the incidence of PTDM. Corticosteroids (administered at high dosages as part of the induction therapy and/or for treating acute rejection and as main constituent of the maintenance immunosuppressive protocol) and calcineurin inhibitors (CNIs; Tacrolimus and Cyclosporine A) are the main responsible for this complication, while the diabetogenic effects of mammalian target of rapamycin inhibitors (mTOR-Is) are still debated (21).

## Main biological factors potentially involved in diabetogenic effects of the mTOR-inhibitors

mTOR inhibitors (mTOR-Is, Sirolimus, and Everolimus) exert their pharmacological effects via inhibition of the serine/threonine kinase mammalian target of rapamycin (mTOR). mTOR integrates signals from growth factors, hormones, nutrients, and cellular energy levels to regulate protein translational and cell growth, proliferation, and survival (22). mTOR exists in two complexes, mTOR complex 1 (mTORC1) and mTOR complex 2 (mTORC2) (23).

mTORC1 also includes RAPTOR (24, 25), MLST8 (26), PRAS40 (27), and DEPTOR, while mTORC2 includes RICTOR, MAPKAP1, PRR5/PRR5L, Mlst8, and Deptor (28–30).

This complex is less sensitive to the acute treatment with mTOR-Is, while long-term administration of these medications inhibits mTORC2 activity by acting on complex integrity (31, 32).

The use of mTOR-Is as immunosuppressants in solid organ transplantation is mostly based on their ability to block cell cycle progression from G1 to the S phase and cellular proliferation (33). Additionally, these drugs may have further therapeutic effects by modifying: (a) protein synthesis, (b) cell cycle, (c) lipid metabolism, (d) energy metabolism, (e) autophagy, (f) angiogenesis, (g) cytoskeleton remodeling, and (h) epithelial to mesenchymal transition (23, 34–39).

Because of their specific pharmacological characteristics, mTOR-Is are highly effective in renal transplantation, and owing to their relative lack of nephrotoxicity, these inhibitors are a valid alternative to CNIs for the maintenance of immunosuppression in renal transplant recipients with chronic allograft nephropathy (40).

Although the clinical utility of this drug category is clear, as other immunosuppressive drugs, mTOR-Is may persuade the development of some adverse effects that need to be immediately recognized and treated to avoid severe illness in kidney transplant recipients.

Numerous clinical and translational studies have highlighted that mTOR-Is-treated kidney transplant recipients may develop metabolic disorders, including PTDM (41–43). As proven in *in vitro* and in animal models, mTOR-Is may decrease beta-cell mass through an increment of the rate of apoptosis (44–47), induce impairment of glucose-induced insulin secretion (45–49), and facilitate glucose intolerance and insulin resistance (50–52) (Table 1).

**Table 1:** mTOR-Is-mediated biological mechanisms involved in glucose dysmetabolism.

Experimental model		mTOR-I	mTOR-I dose	Treatment duration	Main results	References
*In vitro*	Pancreatic beta-cell line from mouse (MIN-6)	Rapamycin	200 nmol/L	24–72 h	Increase in apoptosis, decrease in beta-cell size, and reduction in both basal and glucose-stimulated insulin secretion, likely to be caused by the inhibition of Akt mediated by the inactivation of mTORC2	(46)
	Pancreatic beta-cell line from hamster (HIT-T15)	Sirolimus	0–100 ng/mL	48 h	Reduction in glucose-stimulated insulin secretion	(49)
	Pancreatic beta-cell line from mouse (MIN-6)	Rapamycin	10–100 nmol/L	19 h	Increase in apoptosis	(47)
	Rat L6 myotubes	Rapamycin	10–100 nM	48 h	Reduction in basal and insulin-stimulated glucose uptake and glycogen synthesis. Rapamycin prevents almost completely the translocation of GLUT4 to the plasma membrane following insulin stimulation. All these effects seem to be mediated by the prevention of insulin-induced Akt activation	(51)
	C2C12 myotubes	Rapamycin	500 nM	24 h	Disruption of mTORC2 complex that causes insulin resistance	(53)
*Ex vivo*	Human and rat pancreatic islets	Rapamycin	100 nmol/L	4 days	Reduction in glucose-stimulated insulin secretion and increase in apoptosis	(47)
	Human and rat pancreatic islets	Rapamycin	200 nmol/L	24 h	Reduction in glucose-stimulated insulin secretion and increase in apoptosis mediated by the inactivation of mTORC2	(46)
	PBMC of 30 kidney transplant recipients who were treated with rapamycin for 6 months	Rapamycin	Trough level 8 to 12 ng/mL		Decrease in basal and insulin-stimulated Akt phosphorylation which correlated with the increase of patients’ insulin resistance. Rapamycin inhibits insulin-induced tyrosine phosphorylation of IRS-1	(52)
	Adipocytes obtained via subcutaneous and omental fat biopsies in human donors.	Rapamycin	0.01 uM	15 min or 20 h	Decrease in basal and insulin-stimulated glucose uptake. This effect may be mediated by decreased mTORC2 assembly, AKT Ser473 and AS160 Thr642 phosphorylation. Moreover, rapamycin reduces IRS-2 protein level.	(54)
	Islets of Langerhans isolated from Wistar rats	Rapamycin	30 nM	24 h	Reduction of high glucose-induced insulin secretion mediated by low mitochondrial ATP production through a reduced a-ketoglutarate dehydrogenase activity that limits the velocity of carbohydrate metabolism in the Krebs cycle.	(55)
	Islets isolated from C57BL/6 mice	Rapamycin	1 or 10 ng/mL	24 h	Apoptosis of beta-cells and reduction of insulin production through overinduction of autophagy	(56)
*In vivo*	Wistar rats	Sirolimus	2mg/kg/day	3 weeks	Impairment of glucose tolerance and muscle insulin resistance by preventing full insulin-induced Akt activation and altering the expression and translocation of glucose transporters to the plasma membrane	(51)
	*Psammomys obesus* mice	Rapamycin	0.2 mg/kg/day	2 weeks	Reduction in Akt phosphorylation, increase in glycogen synthase kinase 3β (GSK3) and c-Jun NH2-terminal kinase (JNK) activities in muscle and islets that may account for rapamycin-induced insulin resistance and cell apoptosis	(41)
	C57BL/6 mice	Rapamycin	2 mg/kg/day	14–28 days	Disruption of mTORC2 complex that blocks its inhibiting activity on hepatic gluconeogenesis	(57)
	Sprague–Dawley rats	Rapamycin	2 mg/kg/day	15 days	Glucose intolerance mediated by increased hepatic gluconeogenesis. Rapamycin induces the upregulation of gluconeogenic genes, PEPCK and G6Pase, transcriptional co-activator PGC1-a, and enhances the nuclear recruitment of FoxO1, CRTC2, and CREB.	(58)
	Pancreatic b cells of green fluorescent protein–microtubule-associated protein 1 light chain 3 transgenic mice	Rapamycin	0.2 mg/kg/day	1, 2, 3, 4 or 5 weeks	Overinduction of autophagy and this effect impaired *in vivo* glucose tolerance until 2 weeks after treatment	(56)

PBMC, peripheral blood mononuclear cell; CREB, cAMP response element-binding protein; CRTC2, CREB-regulated transcription coactivator 2; PEPCK, phosphoenolpyruvate carboxykinase; G6Pase, glucose-6-phosphatase; PGC1-a, transcriptional co-activator PPARg coactivator-1a; FoxO1, forkhead box O1.

The impact of mTOR-Is on glucose metabolism is also mediated by its interference with insulin signal transduction. Physiologically, insulin and insulin-like growth factors (IGF) activate mTORC1 through the IRS/PI3K/Akt pathway (50). The mTOR-Is bind to mTOR and suppress the PI3K/AKT pathway (23). The reduction of Akt phosphorylation causes an increment in beta-cell apoptosis with consequent reduced beta-cell mass, impairment of glucose-stimulated insulin secretion, and proinsulin biosynthesis (48). This leads to augmented gluconeogenesis (58), reduced glucose uptake (54), glycolysis, and glycogen synthesis (41, 59) (Table 1).

However, these properties seem to depend on the metabolic context in which they are studied. mTOR-I administered to an animal model of nutrition-dependent type 2 diabetes (diabetic *Psammomys obesus*) worsened the metabolic state of the diabetic animals: augmented insulin resistance, β-cell dysfunction, and death, thereby preventing β-cell adaptation to hyperglycemia (41).

Hyperglycemia and glucose intolerance after mTOR-Is treatment is due to the upregulation of several gluconeogenic genes in the liver via the coordinated activation of peroxisome proliferator-activated receptor-gamma coactivator (PGC)-1alpha, cAMP response element-binding protein (CREB), CREB-regulated transcription coactivator 2 (CRTC2), and forkhead box O1 (FoxO1) (58).

Reduced obesity and hyperlipidemia are frequently associated with the mTOR-I treatment (58, 60). Both *in vitro* and *in vivo* studies have revealed the association between adipogenesis and the mTOR pathway (61–64). Notably, obesity and overnutrition trigger chronic hyperactivation of mTOR activity in multiple tissues (50, 61). In humans, increased S6K activity (65, 66) and overphosphorylation of translation suppressor 4EBP have been observed in obesity (67). Furthermore, accelerated adipogenesis and obesity have been reported in mice lacking *4E-BP1* and *4E-BP2* genes (68). Indeed, targeting the mTOR pathway has been suggested as a treatment for obesity. Consistently, S6K knockout mice were protected against obesity due to increased lipolysis levels and energy expenditure (61, 69, 70).

mTOR-Is treatment may, then, reduce the cell number in adipose tissue with a small contribution from reduced adipocyte size. Reduced lipid uptake and fat cell number impairs the capacity of adipose tissue for plasma lipid clearance, which likely contributes to hyperlipidemia (58).

The inhibition of the mTOR pathway may also play a role in insulin secretion in pancreatic β-cells. Several studies have reported the inhibition of glucose-induced insulin secretion in clonal β-cell lines and in islets (41), but the exact mechanism is unclear (44).

The control of insulin secretion in beta-cells involves several signaling pathways. One proposed mechanism is that inhibition of mTORC1 decreases mitochondrial function, specifically, the activity of α-ketoglutarate dehydrogenase. This results in reduced carbohydrate metabolism and therefore, reduced mitochondrial ATP production (55), which regulates insulin secretion in β-cells (71). Other explanation that rapamycin promotes autophagy, and the intracellular degradation of cytoplasmic proteins involved in the insulin production and secretion (56).

Moreover, several *in vitro* studies have reported a leading role of mTORC2 in the function and survival of beta-cells (46, 72) and insulin resistance (53). Chronic treatment with mTOR-I also inactivates mTORC2 with subsequent inhibition of AKT signaling (46) and other mTORC2 substrates such as protein kinases PKCα, SGK1 substrate NDRG1 in the liver, muscle, and white adipose tissue (57, 73).

It has also been observed that long-term treatment with mTOR-Is (20 weeks) partially restored the detrimental effects on metabolism with enhanced insulin sensitivity, increased oxygen consumption, and improved serum lipid profile with a certain degree of glucose intolerance (74).

Furthermore, in several maintenance immunosuppressive therapeutic protocols, mTOR-I are combined with CNIs.

Numerous observations have reported that CNIs treatment may lead to PTDM by a multifactorial mechanism, which includes impaired insulin secretion, insulin resistance, altered glucokinase function, mitochondrial impairment, and pancreatic β-cell apoptosis (75–79).

The mechanism in pancreatic insulin-secreting β cells seems to be mediated by the inhibition of two targets of calcineurin: nuclear factor of activated T-Lymphocytes (NFAT) and cAMP responsive element binding protein (CREB) (80). Both transcription factors mediate the expression of IRS-2 which promotes β-pancreatic cell growth, proliferation, and survival, insulin secretion by mediating phosphorylation of Akt in response to insulin and insulin-like growth factor (IGF)-1 (81). Through the inhibition of these signaling pathways, CNIs diminish β-cell survival and replication and promote β-cell failure (81–84).

Additionally, pancreatic islets treated with CNIs showed significant morphological alterations in the form of cytoplasmic swelling and vacuolization, degranulation, and immunohistochemical and ultrastructural loss of secretory granules (85, 86).

Tacrolimus (TAC) appears also to reduce insulin secretion through a downregulation of the production of ATP and glycolysis due to a reduced activity of glucokinase, a rate-limiting enzyme in glycolysis that represents an important glucose sensor in pancreatic β-cells (78).

The diabetogenic effect of TAC can also be enhanced by mitochondrial dysfunction through a decrease in both mitochondrial respiration activity and mitochondrial mass (87).

Most studies comparing the diabetogenic effects of the CNIs report higher rates of PTDM among patients receiving TAC compared to cyclosporine A (CsA) (88–90).

## Diabetogenic impact of mTOR-Is: main clinical aspects

During the last two decades, mTOR-Is (mainly Everolimus) have been widely used as part of the maintenance immunosuppressive therapy of kidney transplant recipients and clinical studies/trials have investigated their possible pathogenetic impact on PTDM (Table 2).

**Table 2:** List of major clinical trials investigating the impact of mTOR-I on PTDM.

mTOR-I treatment	Drug	No of patients	Study design	Follow-up time	PTDM outcome and study conclusion	References
*De Novo*	Sirolimus	Prior to transplantation, patients were randomized to one of four treatment groups: Standard-dose CsA (390 patients) Low-dose CsA (399 patients) Low-dose TAC (399 patients) Low-dose SRL (401 patients)	*Standard-dose CsA*: CsA TL 150–300 ng/mL for the first 3 months and 100–200 ng/mL thereafter. *Low-dose CsA*: CsA TL 50–100 ng/mL *Low-dose TAC*: TAC TL 3–7 ng/mL *Low-dose SRL*: SRL TL 4–8 ng/mL All groups received oral MMF (2 g/day) and corticosteroid (5 mg/day).	1 year, 3 years	Incidence of PTDM after 1 year of follow-up was 6% in the standard-dose CsA Group, 4.2% in the low-dose CsA Group, 8.4% in low-dose TAC Group, and 6.6% in low-dose SRL Group (*p* = 0.02 for all comparisons). After 3 years of follow-up the incidence of PTDM was 8% in the standard-dose CsA Group, 5% in the low-dose CsA Group, 12% in low-dose TAC Group, and 8% in low-dose SRL Group. Most PTDM patients did not require long-term antidiabetic medication. The increment of PTDM after the first year was less than 1% although the patients have been exposed to the drug for another 2 years.	(91, 92)
	Sirolimus	Prior to transplantation, patients were randomized to one of two groups: SRL Group (71 patients) CsA Group (74 patients)	*SRL group*: patients began SRL within 48 h after transplantation loading dose of 15 mg for 2 days after transplantation followed by 10 mg/day, then adapted to maintain TL between 10 and 15 ng/mL. *CsA group*: during the first 3 months after transplantation, TL were targeted between 150 and 250 ng/mL reducing to between 75 and 150 ng/mL from the 4th month onward. All patients received a 5-day course of ATG and corticosteroids for the first 6 months and oral MMF (2 g/day) throughout the study.	1 year, 5 years	After 1 year of follow-up, the incidence of PTDM was 9% in the SRL Group and 3% in the CsA Group (*p* = 0.07). In the 5 years follow-up, 2 patients developed PTDM in SRL Group and 4 in CsA Group. Higher incidence of PTDM in the SRL group in the first year after transplantation (p = 0.07) but not in the follow-up (*p* = 0.69).	(93, 94)
	Sirolimus	Patients were randomized before transplantation to one of two groups: CsA Group (38 patients) SRL Group (40 patients)	*SRL group*: SRL TL 30 ng/mL for 2 months, and 15 ng/mL thereafter. *CsA group*: CsA TL 200–400 ng/mL for 2 months, and 100–200 ng/mL until the end of the study. All patients received corticosteroids (5–10 mg/day from month 6 to month 12) and MMF 2 g/day.	1 year	1 patient developed PTDM in the SRL Group and 1 in the CsA Group. No difference in the incidence of PTDM between the 2 study groups.	(95)
	Sirolimus	*De novo* renal allograft recipients were randomly assigned to one of three treatment groups: SRL + TAC-Elim (152 patients) SRL + MMF (152 patients) TAC + MMF (139 patients)	*SRL ± TAC-Elim group*: within 48 h after transplantation, patients received a loading dose of SRL up to 15 mg, followed by 5 mg/day to maintain TL of 8–15 ng/mL through week 13, then 12–20 ng/mL after TAC elimination. TAC was initiated within 24 h of transplantation with a dose up to 0.2 mg/kg/day (in divided doses) to maintain TL of 6–15 ng/mL through week 13, then decreased by 25% per week until fully eliminated. *SRL ± MMF group*: loading dose of up to 15 mg followed by 5 mg/day of SRL was initiated within 48 h after transplantation. The initial target TL of SRL were 10–15 ng/mL through week 26, and 8–15 ng/mL thereafter. MMF 1-2 g/day. *TAC ± MMF group*: oral dose of up to 0.2 mg/kg/day of TAC was initiated within 24 h of transplantation. Target TL were 8–15 ng/mL through week 26 and 5–15 ng/mL thereafter. MMF 1-2 g/day. All patients received CS tapered to 5 mg/day.	1 year	The incidence of PTDM was 22.5% in SRL + TAC-Elim Group, 6% in SRL + MMF Group and 10.9% in TAC + MMF Group. The incidence of PTDM was significantly less in SRL + MMF Group compared with those recipients receiving TAC (*p* = 0.004).	(96)
Switch from CNI to mTOR-I	Conversion from CsA to SRL	Group I: 26 patients converted to SRL Group II: 15 patients who were treated with TAC + SRL for the first 3 months after grafting and thereafter with SRL alone	*Group I*: CsA-treated patients who received the histologic diagnosis of chronic allograft nephropathy (CAN) with serum creatinine levels < 2.5 mg/dL and daily proteinuria ≤ 1.0 g, were converted from CsA to SRL (TL 8–12 ng/mL), low-dose steroids (prednisone 2.5 to 5 mg/day) and MMF (1–2 g/day). *Group II*: patients receiving TAC (TL 6–8 ng/mL), SRL (TL 4–8 ng/mL), and low-dose steroids for the first 3 months after grafting underwent abrupt discontinuation of TAC whereas SRL daily dose was increased to achieve TL 8–12 ng/mL. All patients underwent an oral glucose tolerance test and intravenous insulin tolerance test before and 6 months after the conversion to SRL-alone therapy.	6 months	The withdrawal of CsA or TAC was associated with a significant fall of insulin sensitivity and with a defect in the compensatory beta-cell response. The switch to SRL was associated with a 30% increase of incidence of impaired glucose tolerance and with four patients’ developing PTDM. SRL increased peripheral insulin resistance and impaired pancreatic beta-cell response.	(97)
	Conversion from CsA to SRL	3 months after transplantation patients were randomized to one of two groups: SRL Group (95 patients) CsA Group (97 patients)	*SRL group*: conversion from CsA to SRL 3 months after transplantation. TL was maintained to 8–15 ng/mL until 39 weeks and lowered to 5–10 ng/mL until the end of the study. CsA Group: patients remained on CsA-based immunosuppression (TL 500–800 ng/mL). All patients received oral MMF (2 g/day) and steroids until month 8.	1 year	3 patients developed PTDM in the SRL Group and 2 in the CsA Group. The early conversion from a CsA-based to SRL-based immunosuppression did not induce PTDM	(98)
	Conversion from CsA to EVR	4.5 months after transplantation patients were randomly assigned to one of two groups: EVR Group (155 patients) CsA Group (145 patients)	*EVR group*: 4.5 months after transplantation CsA was replaced with EVR (TL 6–10 ng/mL). *CsA group*: from months 4·5–6 after transplantation, C-0 h (and C-2 h) targets were 120–180 ng/mL (700–1,000 ng/mL), and after month 6, 100–150 ng/mL (500–800 ng/mL). All patients received MMF (1,440 mg/day) and corticosteroids (≥5 mg/day).	1 year	Three patients developed PTDM in the CsA Group and 2 in EVR Group. The early conversion from a CsA-based to SRL-based immunosuppression did not induce PTDM	(99)
	Conversion from CsA or TAC to SRL	SRL Group:555 patients CNI Group: 275 patients	*SRL group*: from 6 to 120 months posttransplant patients were converted to SRL (TL 8–20 ng/mL) *CNI group*: patients remained on CsA-(TL 50–250 ng/mL) or TAC-(TL 4–10 ng/mL) based immunosuppression. Both groups received corticosteroid (2.5 to 15 mg/day)	2 years	Incidence of PTDM was 4.7% in the SRL Group and 4.4% in the CNI Group. The frequency of PTDM was not significantly different between SRL conversion and CNI continuation groups	(100)
	Conversion from CsA or TAC to EVR	Patients at least 6 months after transplantation were randomized to one of two groups: CNI elimination Group (127 patients) CNI minimization Group (144 patients) Control Group (123 patients)	*CNI elimination group*: patients were converted to EVR (8–12 ng/mL) with CNI elimination. *CNI minimization group*: patients were converted to EVR (3–8 ng/mL) with CNI minimization by 20%. *Control group*: patients continued CNI without changes in TL.	2 years	Incidence of PTDM was 4.7% in the CNI elimination group (*p* = 0.75 vs. Control), 4.9% in the CNI minimization Group (*p* = 0.55 vs. Control) and 3.3% in the Control Group.	(101)
	Conversion from CNI to SRL after development of PTDM	Patients with a diagnosis of PTDM were divided into two groups: CNI Group (8 patients) SRL Group (21 patients)	*CNI group*: CNIs were reduced to achieve TL of 5–7 ng/mL (TAC) and 130–150 mg/dL (CsA). *SRL group*: CNI (TAC or CsA) was converted to SRL (TL 7–10 ng/mL). MMF was given at a dose of 1 to 2 g/day. Prednisolone 5 mg/day by the end of 6th month.	5 years	PTDM resolved in 37.5% of CNI Group and in 80% of SRL Group. The conversion from CNI to SRL could improve significantly the metabolic parameters of patients with PTDM.	(102)
mTOR-I + CNI	EVR + CsA	Within 48 h after transplantation, patients were randomly assigned to one of the following Groups: EVR 1.5 mg/day Group (194 patients) EVR 3 mg/day Group (198 patients) MMF Group (196 patients)	*EVR 1.5 mg/day group*: EVR 1.5 mg/day + CsA (150–400 ng/mL during weeks 1–4 and 100–300 ng/mL thereafter) and prednisone (5 mg/day). *EVR 3 mg/day group*: EVR 3 mg/day + CsA (150–400 ng/mL during weeks 1–4 and 100–300 ng/mL thereafter) and prednisone (5 mg/day). *MMF group*: received oral MMF (2 g/day) + CsA (150–400 ng/mL during weeks 1–4 and 100–300 ng/mL thereafter) and prednisone (5 mg/day).	3 years	Incidence of PTDM was 12.6% in patients receiving 3 mg/day EVR, 6.7% in patients receiving 1.5 mg/day EVR and 5.6% in patients receiving MMF. Although not statistically significant high dosage EVR was associated with a higher incidence of PTDM	(103)
	SRL + TAC	Patients were assigned to one of three treatment groups: TAC-SRL 0.5 mg Group (325 patients) TAC-SRL 2 mg Group (325 patients) TAC-MMF Group (327 patients)	*TAC-SRL 0.5 mg group*: TAC whole blood TL of 8–16 ng/mL between days 0 and 14, and 5–15 ng/mL between days 15 and 183. Corticosteroid 5 mg/day. MMF 1 g/day. SRL: 0.5 mg/day. *TAC-SRL 2 mg group*: TAC whole blood TL of 8–16 ng/mL between days 0 and 14, and 5–15 ng/mL between days 15 and 183. Corticosteroid 5 mg/day. MMF 1 g/day. SRL: 2 mg/day. *TAC-MMF group*: TAC whole blood TL of 8–16 ng/mL between days 0 and 14, and 5–15 ng/mL between days 15 and 183. 1 g/day MMF.	6 months	Incidence of PTDM was 6.8% in patients receiving 0.5 mg SRL, 15.2% in patients receiving 2 mg SRL and 9.5% in the TAC-MMF group (*p* = 0.005, Fisher’s exact test). The number of patients requiring insulin for PTDM was similar in the TAC-SRL 2 mg and TAC-MMF treatment groups	(104)
	SRL + TAC	TAC/SRL Group: 318 patients TAC-MMF Group: 318 patients	*TAC/SRL group*: TAC TL 4–8 ng/mL on days from 15 to 42 and 4–6 ng/mL on days from 43 to 183. SRL dose: 2.0 mg for 28 days and 1.0 mg thereafter. *TAC-MMF group*: TAC TL 8–12 ng/mL on days from 15 to 42 and 5–10 ng/mL on days from 43 to 183. MMF: 2.0 g for the first 14 days and 1.0 g daily thereafter. Steroids were to be steadily tapered from 20 mg on day 2 to 5 mg by day 90 and discontinued on day 91.	6 months	The incidence of PTDM was lower in the TAC/SRL than in the TAC/MMF group. Patients requiring antidiabetic treatment was 24.8% in TAC/MMF Group and 17.8% in TAC/SRL Group.	(105)
	SRL + CsA or TAC	Immediately before transplantation patients were randomized into one of three study groups: TAC/SRL Group (50 patients) TAC/MMF Group (50 patients) CsA/SRL Group (50 patients)	*TAC/SRL group*: TAC TL was lowered to 6–10 ng/mL between 3 to 6 month post-transplant and 4–8 ng/mL thereafter. SRL TL: 6–10 ng/mL *TAC/MMF group*: TAC TL was lowered to 6–10 ng/mL between 3 to 6 month post-transplant and 4–8 ng/mL thereafter. MMF dose 2 g/day *CsA/SRL group*: CsA was initiated at 5 mg/kg twice daily with an initial target trough level of 200–250 ng/mL, then lowered to 100–200 ng/mL thereafter. SRL TL: 6–10 ng/mL	8 years	Incidence of PTDM was 19% in TAC/MMF Group, 32% in TAC/SRL Group and 31% in CsA/SRL Group. The rate of developing PTDM was not significantly different among the three groups	(106)
	SRL + TAC	Before transplantation patients were randomized into one of two groups: TAC/SRL Group (37 patients) TAC-MMF Group (45 patients)	*TAC/SRL group*: Target 12-h trough levels for TAC were 8–10 ng/mL during the first 3 months, 7–9 ng/mL from 4 to 6 months post-transplant and 6–8 ng/mL thereafter. SRL TL: 7–10 ng/mL *TAC-MMF group*: Target 12-h trough levels for TAC were 8–10 ng/mL during the first 3 months, 7–9 ng/mL from 4 to 6 months post-transplant and 6–8 ng/mL thereafter. MMF dose: 2 g/day	3 years, 8.5 years	The incidence of PTDM after 3 years of follow-up was: 5% in TAC/SRL Group and 3% in TAC/MMF Group. After 8.5 years of follow-up 24.3% of patients in the TAC/SRL Group and 13.3% in the TAC/MMF Group developed PTDM (*p* = 0.25). The rate of developing PTDM was not significantly different between the two groups	(107, 108)
	EVR + CsA	Within 24 h post-transplantation patients were randomized into one of three groups: EVR 1.5 mg Group (277 patients) EVR 3.0 mg Group (279 patients) MPA Group (277 patients)	*EVR 1.5 mg group*: EVR TL 3–8 ng/mL + reduced exposure CsA *EVR 3 mg group*: EVR TL 6–12 ng/mL + reduced exposure CsA *MPA group*: MPA dose 1.44 g + standard-exposure CsA CsA administered according to TL	1 year	The incidence of PTDM was similar in all groups: 5.1% in the EVR 1.5 mg Group, 7.9% in the EVR 3.0 mg Group and 7.0% in the MPA Group.	(109)
	SRL + CNI and MMF	CSA + MMF/AZA Group (9,095 patients) TAC + MMF/AZA Group (8,431 patients) SRL MMF/AZA Group (619 patients) SRL/CSA Group (800 patients) SRL/TAC Group (1,179 patients)	The data source for the study was the United States Renal Data System		The 3-year cumulative incidence of PTDM in patients treated with SRL/CsA and with SRL/TAC was 21.9 and 21.5%, respectively. Patients treated with TAC and MMF/AZA had the next highest incidence of PTDM (cumulative incidence 19.0%). Patient treated with SRL and MMF/AZA had a cumulative incidence of PTDM of 17.8%. Patients treated with CsA in combination with MMF/AZA had the lowest incidence of PTDM (15.6%; overall log rank *p* < 0.0001)	(42)
	EVR + CNI	Within 24 h of transplantation patients were randomized into one of two groups: EVR + reduced-exposure CNI (rCNI) Group (1,022 patients) MPA + standard-exposure CNI (sCNI) Group (1,015 patients)	*EVR ± rCNI group*: EVR TL: 3–8 ng/mL; TAC 4–7 ng/mL during months 0–2, 2–5 ng/mL during months 3–6, and 2–4 ng/mL thereafter; CsA 100–150 during months 0–2, 50–100 during months 3–6, and 25–50 ng/mL thereafter *MPA ± sCNI group*: MPA was given as enteric-coated mycophenolate sodium (1.44 g/day) or MMF (2.0 g/day), which could be reduced after week 2 to enteric-coated mycophenolate sodium 1.08 g/day or MMF 1.5 g/day in patients receiving TAC but not those given CsA The tacrolimus dose was adjusted to target C0 concentrations of 8–12 ng/mL during months 0–2, 6–10 ng/mL during months 3–6, and 5–8 ng/mL thereafter; corresponding target ranges for CsA were 200–300, 150–200, and 100–200 ng/mL, respectively. All patients received corticosteroid dose minimum 5 mg/day	2 years	Incidence of PTDM was similar in both groups (19.6% vs. 18.6%)	(110)

CsA, Cyclosporine A; SRL, Sirolimus; TL, trough level with TAC, Tacrolimus; MMF, Mycophenolate Mofetil; CNI Calcineurin inhibitor; AZA, Azathioprine; EVR, Everolimus; MPA, mycophenolic acid.

As reported by the SYMPHONY study after 1 year and 3 years of follow-up (91, 92), patients treated with low-dose sirolimus (SRL) plus Mycophenolate Mofetil (MMF) presented a higher incidence of PTDM than those treated with low dose of CsA plus MMF (6.6% vs. 4.2% after the first year and 8% vs. 5% after 3 years of follow-up). No differences were observed in the comparison between the standard dose of CsA vs. low dose SRL (in both groups the incidence was 8%). This study suggested that a low-dose CsA-based maintenance immunosuppressive treatment or a switch from TAC to low dose of CsA or SRL could be beneficial for kidney transplant recipients at high risk of PTDM. However, in our opinion, this therapeutic strategy should be undertaken only in highly selected patients after weighing the risk of rejection or in the absence of additional adverse events. In the prospective randomized SPIESSER study, which compared the safety and efficacy of a SRL plus MMF-based immunosuppressive regimen with a CsA plus MMF-based regimen after an induction therapy with polyclonal antilymphocyte antibodies and withdrawal of steroids at 6 months’ post-transplantation, it was observed a higher incidence of PTDM in the SRL group in the first-year post-transplantation (9% vs. 3%, *p* = 0.07) (93). Instead, no differences were reported between the 2 study groups after 5 years of follow-up (2% vs. 4%, *p* = 0.69) (94). This study revealed potential diabetogenic effects of the mTOR-Is in the early post-transplant phase (probably induced by the high-dosages of these drugs in association with corticosteroids). However, the relatively low number of patients with a diagnosis of PTDM, the similar risk of PTDM between the two study groups, and the high rate of conversion from the randomized immunosuppression to other regimens in the SRL group may not allow reaching a definitive conclusion revealing the need of a larger trial on this specific topic.

Kreis et al. (ORION Study) also described no differences in the incidence of hyperglycemia and insulin-dependent PTDM in patients treated with SRL compared to CsA (95). The evaluation of two SRL-based regimens, one with CNI withdrawal (SRL + TAC-Elim) and the other with complete CNI avoidance (SRL + MMF), compared with a CNI-based regimen containing TAC + MMF in *de novo* renal allograft recipients demonstrated higher incidence of PTDM in TAC recipients confirming a diabetogenic effect of TAC compared to SRL (96). These results could be partially explained by the relatively high trough level of TAC used in this group.

Also in the conversion trials, the switch from CNI to mTOR-I has not shown clear diabetogenic effects. However, some authors have reported an increased risk of PTDM in mTOR-I-treated patients due to a drug-related enhancement of peripheral insulin resistance and impairment of the compensatory beta-cell response (97).

In both CONCEPT and ZEUS studies, the early conversion from CsA-based to SRL-based therapeutic regimen (3 or 4.5 months after transplantation) did not induce PTDM in a 12 months-period post-transplantation (98, 99).

Similarly, in the late conversion (CONVERT) study, where the renal allograft recipients were randomly assigned (2:1) to undergo conversion from CsA- or TAC-based immunosuppression to SRL or to continue receiving CNI-based therapy for 2 years, the frequency of PTDM was similar between the two study regimens (4.7% vs. 4.4%, *p* = 1.000) (100).

Holdaas et al., in the ASCERTAIN study, which included kidney transplant patients with allograft impairment (GFR 30–70 mL/min) who underwent the minimization of CNI or conversion to Everolimus (EVR), reported a percentage of patients with PTDM of 4.7% in the CNI withdrawal group, 4.9% in the minimization group and 3.3% in the control group (101).

All these studies (98–101), although performed using SRL alone in a heterogeneous patients’ population, encourage clinicians to reduce the dosages of this immunosuppressive drug.

Nevertheless, a recent systematic review and meta-analysis established that the conversion from CNIs to mTOR-Is did not significantly decrease the risk of PTDM (111).

Instead, Veroux et al. (102) showed that the conversion from CNI to mTOR-I-based therapy in patients with PTDM had a positive effect on insulin-stimulated glucose uptake. In this study, it was observed an improved glucose balance in 80% of patients converted to SRL compared with those patients (37.5%) in whom a reduction in CNI dose was carried out. No change was found in the incidence of acute rejection. According to these authors, such beneficial effects of mTOR-Is on the glycemic homeostasis could be explained by the chronic inhibition of mTORC1 (a biological/pharmacological effect such as that observed after metformin administration) (112). In this single-center study, the small sample size (particularly of patients treated with mTOR-Is) and the low incidence of PTDM (probably due to an early reduction of immunosuppression) cannot allow to draw any definitive conclusions. In all cases, a conversion from CNIs to mTOR-Is should be achieved only after a carefully benefit–risk evaluation.

Unfortunately, also the impact of the combined therapy of mTOR-Is plus CNIs on PTDM is still argued.

Vitko et al. in a 36-month, multicenter, randomized, parallel-group equivalence trial of two oral doses of EVR (1.5 or 3 mg/day) vs. MMF (2 g/day) along with CsA microemulsion (Neoral) and corticosteroids in *de novo* renal transplant recipients, reported a higher incidence of PTDM in patients receiving 3 mg/day EVR (12.6%) compared to those receiving a low dose of EVR (6.7%) and MMF (5.6%) (103).

In another 6-month, randomized, open-label, parallel-group, comparative trial comparing two regimens of TAC plus SRL (with either 0.5 or 2 mg) with a TAC plus MMF immunosuppressive schema, authors found that a larger number of patients treated with TAC plus SRL at 2 mg developed PTDM (*p* = 0.005). However, the number of patients requiring insulin for PTDM was similar in the TAC/SRL 2 mg and TAC/MMF groups (*p* > 0.05) (104).

In a multicenter trial, in which renal transplant recipients were randomized to TAC with fixed-dose SRL (*N* = 318) or TAC with MMF (*N* = 316), 6 months’ creatinine clearance was comparable between the 2 immunosuppressive schemas. Biopsy-confirmed acute rejection was 15.1% (TAC/SRL) and 12.3% (TAC/MMF). In both groups, graft survival was 93% and patient survival was 99%. Premature withdrawal due to an adverse event was twice as high in the TAC/SRL group (15.1% vs. 6.3%). The incidence of any antidiabetic treatment for >30 consecutive days in previously nondiabetic patients was 17.8% in TAC/SRL, and 24.8% in TAC/MMF (105).

Guerra et al., have, then, studied a long-term follow-up post-transplant (8 years) to compare TAC/SRL, TAC/MMF, and CsA/SRL. In this report, the incidence of PTDM was not significantly different among the three groups (*p* = 0.37), while a slightly smaller percentage of PTDM was registered in the TAC/MMF group (19%) than TAC/SRL (32%) and CsA/SRL (31%) group. However, difference did not reach any statistical difference (*p* = 0.16) (106). These results were confirmed by Gallon et al. and by Chhabra et al. (107, 108).

Moreover, in a 24-month, open-label study, 833 *de novo* renal-transplant recipients were randomized to EVR 1.5 or 3 mg/day (target troughs 3–8 and 6–12 ng/mL, respectively) with reduced-exposure CsA, or mycophenolic acid (MPA) 1.44 g/day plus standard-exposure CsA. The overall incidence of PTDM and adverse events were comparable between the groups. Corticosteroids were used in more than 99% of patients in each group during the study, with more than 70% receiving corticosteroids without discontinuation throughout the 24-month study period (109). These studies (104–109) demonstrated a similar impact of the combined therapy of CNIs and mTOR-Is on PTDM.

Johnston et al. demonstrated that the incidence of PTDM was 21.9% in patients treated with a combination of SRL plus CsA, 21.5% in those treated with SRL plus TAC, and 17.8% in the group of patients received SRL plus MMF/AZA, showing that rapamycin was an independent variable involved in the development of PTDM (42). This study, although performed on a large dataset, has major limitations including the inherent limitations of retrospective analyses of administrative data sets, the absence of information regarding the dosage of SRL and CNI used, the enrolment of patients who had Medicare as the primary payer, which may limit the applicability of its findings to other patient populations.

In the recent TRANSFORM (Transplant efficacy and safety outcomes with an EVR-based regimen) study, a 24-month, prospective, open-label trial in 2037 *de novo* renal transplant recipients randomized (1:1) within 24 h of transplantation to receive EVR with reduced-exposure CNI (EVR + rCNI) or mycophenolate with standard-exposure CNI, the incidence of PTDM was similar in both the arms (19.6% vs. 18.6%) (110). Even if PTDM was not included as a primary endpoint, this study confirmed the no specific diabetogenic effects of the combined therapy with CNIs plus mTOR-Is (particularly administered at low dosages), as previously suggested. Finally, a recent network meta-analysis involving 206 eligible studies that identified 75,595 patients on TAC, 51,242 on CsA, and 3,020 on SRL, demonstrated that TAC tended to exhibit higher diabetogenicity in the short-term (2–3 years post-transplant), whereas SRL exhibits higher diabetogenicity in the long-term (5–10 years post-transplant) (113). This study is quite difficult to interpreter due to the clinical heterogeneity of the immunosuppression protocols utilized in the included studies (such as co-treatments and therapy used for rejections), variability of the criteria used to define PTDM, and absence of control of several clinical/therapeutic confounding factors.

## Conclusion

After reviewing the available literature on this topic area, we cannot draw any definite conclusions about the diabetogenic impact of the mTOR-Is. However, we can encourage clinicians to lower the dose of these immunosuppressive drugs in patients at high risk of PTDM. Moreover, our paper shows that the transplant scientific community should undertake more research programs to better study this important topic.

## Author contributions

SG, SM, DT, and GZ searched the literature and wrote the manuscript. GZ, GS, and LG revised the manuscript. All authors contributed to the article and approved the submitted version.

## Conflict of interest

The authors declare that the research was conducted in the absence of any commercial or financial relationships that could be construed as a potential conflict of interest.

## Publisher’s note

All claims expressed in this article are solely those of the authors and do not necessarily represent those of their affiliated organizations, or those of the publisher, the editors and the reviewers. Any product that may be evaluated in this article, or claim that may be made by its manufacturer, is not guaranteed or endorsed by the publisher.

## References

[1] Kasiske BL Snyder JJ Gilbertson D Matas AJ. Diabetes mellitus after kidney transplantation in the United States. Am J Transplant. (2003) 3:178–85. doi: 10.1034/j.1600-6143.2003.00010.x 12603213

[2] Davidson J Wilkinson A Dantal J Dotta F Haller H Hernández D. Wheeler DC; international expert panel. New-onset diabetes after transplantation: 2003 international consensus guidelines. Proceedings of an international expert panel meeting. Barcelona, Spain, 19 February 2003. Transplantation. (2003) 75:SS3–SS24. doi: 10.1097/01.TP.0000069952.49242.3E, PMID: 12775942

[3] Sharif A Hecking M de Vries AP Porrini E Hornum M Rasoul-Rockenschaub S. Proceedings from an international consensus meeting on posttransplantation diabetes mellitus: recommendations and future directions. Am J Transplant. (2014) 14:1992–2000. doi: 10.1111/ajt.12850, PMID: 25307034 PMC4374739

[4] Peev V Reiser J Alachkar N. Diabetes mellitus in the transplanted kidney. Front Endocrinol. (2014) 5:141. doi: 10.3389/fendo.2014.00141 PMC4145713 25221544

[5] Yates CJ Fourlanos S Colman PG Cohney SJ. Screening for new-onset diabetes after kidney transplantation: limitations of fasting glucose and advantages of afternoon glucose and glycated hemoglobin. Transplantation. (2013) 96:726–31. doi: 10.1097/TP.0b013e3182a012f3, PMID: 23902993

[6] Shabir S Jham S Harper L Ball S Borrows R Sharif A. Validity of glycated haemoglobin to diagnose new onset diabetes after transplantation. Transpl Int. (2013) 26:315–21. doi: 10.1111/tri.12042, PMID: 23279163

[7] Ponticelli C Favi E Ferraresso M. New-onset diabetes after kidney transplantation. Medicina. (2021) 57:250. doi: 10.3390/medicina57030250, PMID: 33800138 PMC7998982

[8] Weir MR Fink JC. Risk for posttransplant diabetes mellitus with current immunosuppressive medications. Am J Kidney Dis. (1999) 34:1–13. doi: 10.1016/S0272-6386(99)70101-0 10401009

[9] Yang J Hutchinson II Shah T Min DI. Genetic and clinical risk factors of new-onset diabetes after transplantation in Hispanic kidney transplant recipients. Transplantation. (2011) 91:1114–9. doi: 10.1097/TP.0b013e31821620f9, PMID: 21544032

[10] Ahmed SH Biddle K Augustine T Azmi S. Post-transplantation diabetes mellitus. Diabetes Ther. (2020) 11:779–801. doi: 10.1007/s13300-020-00790-5, PMID: 32095994 PMC7136383

[11] Walczak DA Calvert D Jarzembowski TM Testa G Sankary HN Thielke J. Increased risk of post-transplant diabetes mellitus despite early steroid discontinuation in Hispanic kidney transplant recipients. Clin Transpl. (2005) 19:527–31. doi: 10.1111/j.1399-0012.2005.00383.x, PMID: 16008600

[12] Peracha J Nath J Ready A Tahir S Parekh K Hodson J. Risk of post-transplantation diabetes mellitus is greater in south Asian versus Caucasian kidney allograft recipients. Transpl Int. (2016) 29:727–39. doi: 10.1111/tri.12782 27062063

[13] Hotamisligil GS Shargill NS Spiegelman BM. Adipose expression of tumor necrosis factor-alpha: direct role in obesity-linked insulin resistance. Science. (1993) 259:87–91. doi: 10.1126/science.7678183, PMID: 7678183

[14] Stephens JM Lee J Pilch PF. Tumor necrosis factor-alpha-induced insulin resistance in 3T3-L1 adipocytes is accompanied by a loss of insulin receptor substrate-1 and GLUT4 expression without a loss of insulin receptor-mediated signal transduction. J Biol Chem. (1997) 272:971–6. doi: 10.1074/jbc.272.2.971, PMID: 8995390

[15] Rydén M Arner P. Tumour necrosis factor-alpha in human adipose tissue—from signalling mechanisms to clinical implications. J Intern Med. (2007) 262:431–8. doi: 10.1111/j.1365-2796.2007.01854.x, PMID: 17875179

[16] Ryden M Dicker A van Harmelen V Hauner H Brunnberg M Perbeck L. Mapping of early signaling events in tumor necrosis factor-alpha -mediated lipolysis in human fat cells. J Biol Chem. (2002) 277:1085–91. doi: 10.1074/jbc.M109498200, PMID: 11694522

[17] Fabrizi F Messa P Martin P Takkouche B. Hepatitis C virus infection and post-transplant diabetes mellitus among renal transplant patients: a meta-analysis. Int J Artif Organs. (2008) 31:675–82. doi: 10.1177/039139880803100801, PMID: 18825640

[18] Bose SK Shrivastava S Meyer K Ray RB Ray R. Hepatitis C virus activates the mTOR/S6K1 signaling pathway in inhibiting IRS-1 function for insulin resistance. J Virol. (2012) 86:6315–22. doi: 10.1128/JVI.00050-12, PMID: 22457523 PMC3372214

[19] Kasai D Adachi T Deng L Nagano-Fujii M Sada K Ikeda M. HCV replication suppresses cellular glucose uptake through down-regulation of cell surface expression of glucose transporters. J Hepatol. (2009) 50:883–94. doi: 10.1016/j.jhep.2008.12.029, PMID: 19303158

[20] Hjelmesaeth J Müller F Jenssen T Rollag H Sagedal S Hartmann A. Is there a link between cytomegalovirus infection and new-onset posttransplantation diabetes mellitus? Potential mechanisms of virus induced beta-cell damage. Nephrol Dial Transplant. (2005) 20:2311–5. doi: 10.1093/ndt/gfi033 16046502

[21] Kesiraju S Paritala P Rao Ch UM Sahariah S. New onset of diabetes after transplantation—an overview of epidemiology, mechanism of development and diagnosis. Transpl Immunol. (2014) 30:52–8. doi: 10.1016/j.trim.2013.10.006, PMID: 24184293

[22] Wullschleger S Loewith R Hall MN. TOR signaling in growth and metabolism. Cells. (2006) 124:471–84. doi: 10.1016/j.cell.2006.01.016 16469695

[23] Zaza G Granata S Caletti C Signorini L Stallone G Lupo A. mTOR inhibition role in cellular mechanisms. Transplantation. (2018) 102:S3–S16. doi: 10.1097/TP.0000000000001806 29369970

[24] Hara K Maruki Y Long X Yoshino K Oshiro N Hidayat S. Raptor, a binding partner of target of rapamycin (TOR), mediates TOR action. Cells. (2002) 110:177–89. doi: 10.1016/S0092-8674(02)00833-4, PMID: 12150926

[25] Kim DH Sarbassov DD Ali SM King JE Latek RR Erdjument-Bromage H. mTOR interacts with raptor to form a nutrient-sensitive complex that signals to the cell growth machinery. Cells. (2002) 110:163–75. doi: 10.1016/S0092-8674(02)00808-5, PMID: 12150925

[26] Kim DH Sarbassov DD Ali SM Latek RR Guntur KV Erdjument-Bromage H. GbetaL, a positive regulator of the rapamycin-sensitive pathway required for the nutrient-sensitive interaction between raptor and mTOR. Mol Cell. (2003) 11:895–904. doi: 10.1016/S1097-2765(03)00114-X, PMID: 12718876

[27] Thedieck K Polak P Kim ML Molle KD Cohen A Jenö P. PRAS40 and PRR5-like protein are new mTOR interactors that regulate apoptosis. PLoS One. (2007) 2:e1217. doi: 10.1371/journal.pone.0001217, PMID: 18030348 PMC2075366

[28] Jacinto E Loewith R Schmidt A Lin S Rüegg MA Hall A. Mammalian TOR complex 2 controls the actin cytoskeleton and is rapamycin insensitive. Nat Cell Biol. (2004) 6:1122–8. doi: 10.1038/ncb1183 15467718

[29] Pearce LR Huang X Boudeau J Pawłowski R Wullschleger S Deak M. Identification of Protor as a novel Rictor-binding component of mTOR complex-2. Biochem J. (2007) 405:513–22. doi: 10.1042/BJ20070540, PMID: 17461779 PMC2267312

[30] Wälchli M Berneiser K Mangia F Imseng S Craigie LM Stuttfeld E. Regulation of human mTOR complexes by DEPTOR. elife. (2021) 10:e70871. doi: 10.7554/eLife.70871, PMID: 34519268 PMC8439649

[31] Sarbassov DD Ali SM Sengupta S Sheen JH Hsu PP Bagley AF. Prolonged rapamycin treatment inhibits mTORC2 assembly and Akt/PKB. Mol Cell. (2006) 22:159–68. doi: 10.1016/j.molcel.2006.03.029, PMID: 16603397

[32] Huang K Fingar DC. Growing knowledge of the mTOR signaling network. Semin Cell Dev Biol. (2014) 36:79–90. doi: 10.1016/j.semcdb.2014.09.011, PMID: 25242279 PMC4253687

[33] Baroja-Mazo A Revilla-Nuin B Ramírez P Pons JA. Immunosuppressive potency of mechanistic target of rapamycin inhibitors in solid-organ transplantation. World J Transplant. (2016) 6:183–92. doi: 10.5500/wjt.v6.i1.183, PMID: 27011916 PMC4801794

[34] Lupinacci S Perri A Toteda G Vizza D Lofaro D Pontrelli P. Rapamycin promotes autophagy cell death of Kaposi's sarcoma cells through P75NTR activation. Exp Dermatol. (2022) 31:143–53. doi: 10.1111/exd.14438, PMID: 34331820

[35] Zaza G Leventhal J Signorini L Gambaro G Cravedi P. Effects of antirejection drugs on innate immune cells after kidney transplantation. Front Immunol. (2019) 10:2978. doi: 10.3389/fimmu.2019.02978, PMID: 31921213 PMC6930910

[36] Tomei P Masola V Granata S Bellin G Carratù P Ficial M. Everolimus-induced epithelial to mesenchymal transition (EMT) in bronchial/pulmonary cells: when the dosage does matter in transplantation. J Nephrol. (2016) 29:881–91. doi: 10.1007/s40620-016-0295-4, PMID: 27026415

[37] Granata S Dalla Gassa A Carraro A Brunelli M Stallone G Lupo A. Sirolimus and Everolimus pathway: reviewing candidate genes influencing their intracellular effects. Int J Mol Sci. (2016) 17:735. doi: 10.3390/ijms17050735, PMID: 27187382 PMC4881557

[38] Zaza G Granata S Tomei P Masola V Gambaro G Lupo A. mTOR inhibitors and renal allograft: yin and Yang. J Nephrol. (2014) 27:495–506. doi: 10.1007/s40620-014-0103-y, PMID: 24804854

[39] Saxton RA Sabatini DM. mTOR signaling in growth, metabolism, and disease. Cells. (2017) 168:960–76. doi: 10.1016/j.cell.2017.02.004, PMID: 28283069 PMC5394987

[40] Paoletti E Ratto E Bellino D Marsano L Cassottana P Cannella G. Effect of early conversion from CNI to sirolimus on outcomes in kidney transplant recipients with allograft dysfunction. J Nephrol. (2012) 25:709–18. doi: 10.5301/jn.5000044, PMID: 22038336

[41] Fraenkel M Ketzinel-Gilad M Ariav Y Pappo O Karaca M Castel J. mTOR inhibition by rapamycin prevents beta-cell adaptation to hyperglycemia and exacerbates the metabolic state in type 2 diabetes. Diabetes. (2008) 57:945–57. doi: 10.2337/db07-0922, PMID: 18174523

[42] Johnston O Rose CL Webster AC Gill JS. Sirolimus is associated with new-onset diabetes in kidney transplant recipients. J Am Soc Nephrol. (2008) 19:1411–8. doi: 10.1681/ASN.2007111202, PMID: 18385422 PMC2440303

[43] Gyurus E Kaposztas Z Kahan BD. Sirolimus therapy predisposes to new-onset diabetes mellitus after renal transplantation: a long-term analysis of various treatment regimens. Transplant Proc. (2011) 43:1583–92. doi: 10.1016/j.transproceed.2011.05.001, PMID: 21693238

[44] Asahara SI Inoue H Watanabe H Kido Y. Roles of mTOR in the regulation of pancreatic β-cell mass and insulin secretion. Biomol Ther. (2022) 12:614. doi: 10.3390/biom12050614, PMID: 35625542 PMC9138643

[45] Barlow AD Nicholson ML Herbert TP. Evidence for rapamycin toxicity in pancreatic β-cells and a review of the underlying molecular mechanisms. Diabetes. (2013) 62:2674–82. doi: 10.2337/db13-0106, PMID: 23881200 PMC3717855

[46] Barlow AD Xie J Moore CE Campbell SC Shaw JA Nicholson ML. Rapamycin toxicity in MIN6 cells and rat and human islets is mediated by the inhibition of mTOR complex 2 (mTORC2). Diabetologia. (2012) 55:1355–65. doi: 10.1007/s00125-012-2475-7, PMID: 22314813 PMC3328678

[47] Bell E Cao X Moibi JA Greene SR Young R Trucco M. Rapamycin has a deleterious effect on MIN-6 cells and rat and human islets. Diabetes. (2003) 52:2731–9. doi: 10.2337/diabetes.52.11.2731, PMID: 14578291

[48] Ardestani A Lupse B Kido Y Leibowitz G Maedler K. mTORC1 signaling: a double-edged sword in diabetic β cells. Cell Metab. (2018) 27:314–31. doi: 10.1016/j.cmet.2017.11.004, PMID: 29275961

[49] Paty BW Harmon JS Marsh CL Robertson RP. Inhibitory effects of immunosuppressive drugs on insulin secretion from HIT-T15 cells and Wistar rat islets. Transplantation. (2002) 73:353–7. doi: 10.1097/00007890-200202150-00007, PMID: 11884930

[50] Mao Z Zhang W. Role of mTOR in glucose and lipid metabolism. Int J Mol Sci. (2018) 19:2043. doi: 10.3390/ijms19072043, PMID: 30011848 PMC6073766

[51] Deblon N Bourgoin L Veyrat-Durebex C Peyrou M Vinciguerra M Caillon A. Chronic mTOR inhibition by rapamycin induces muscle insulin resistance despite weight loss in rats. Br J Pharmacol. (2012) 165:2325–40. doi: 10.1111/j.1476-5381.2011.01716.x, PMID: 22014210 PMC3413866

[52] Di Paolo S Teutonico A Leogrande D Capobianco C Schena PF. Chronic inhibition of mammalian target of rapamycin signaling downregulates insulin receptor substrates 1 and 2 and AKT activation: a crossroad between cancer and diabetes? J Am Soc Nephrol. (2006) 17:2236–44. doi: 10.1681/ASN.2006030196, PMID: 16807405

[53] Ye L Varamini B Lamming DW Sabatini DM Baur JA. Rapamycin has a biphasic effect on insulin sensitivity in C2C12 myotubes due to sequential disruption of mTORC1 and mTORC2. Front Genet. (2012) 3:177. doi: 10.3389/fgene.2012.00177 22973301 PMC3438685

[54] Pereira MJ Palming J Rizell M Aureliano M Carvalho E Svensson MK. mTOR inhibition with rapamycin causes impaired insulin signalling and glucose uptake in human subcutaneous and omental adipocytes. Mol Cell Endocrinol. (2012) 355:96–105. doi: 10.1016/j.mce.2012.01.024, PMID: 22333157

[55] Shimodahira M Fujimoto S Mukai E Nakamura Y Nishi Y Sasaki M. Rapamycin impairs metabolism-secretion coupling in rat pancreatic islets by suppressing carbohydrate metabolism. J Endocrinol. (2010) 204:37–46. doi: 10.1677/JOE-09-0216, PMID: 19812126

[56] Tanemura M Nagano H Taniyama K Kamiike W Mori M Doki Y. Role of rapamycin-induced autophagy in pancreatic islets. Am J Transplant. (2012) 12:1067. doi: 10.1111/j.1600-6143.2011.03933.x, PMID: 22299600

[57] Lamming DW Ye L Katajisto P Goncalves MD Saitoh M Stevens DM. Rapamycin-induced insulin resistance is mediated by mTORC2 loss and uncoupled from longevity. Science. (2012) 335:1638–43. doi: 10.1126/science.1215135, PMID: 22461615 PMC3324089

[58] Houde VP Brûlé S Festuccia WT Blanchard PG Bellmann K Deshaies Y. Chronic rapamycin treatment causes glucose intolerance and hyperlipidemia by upregulating hepatic gluconeogenesis and impairing lipid deposition in adipose tissue. Diabetes. (2010) 59:1338–48. doi: 10.2337/db09-1324 20299475 PMC2874694

[59] Vergès B Cariou B. mTOR inhibitors and diabetes. Diabetes Res Clin Pract. (2015) 110:101–8. doi: 10.1016/j.diabres.2015.09.014 26421362

[60] Kasiske BL de Mattos A Flechner SM Gallon L Meier-Kriesche HU Weir MR. Mammalian target of rapamycin inhibitor dyslipidemia in kidney transplant recipients. Am J Transplant. (2008) 8:1384–92. doi: 10.1111/j.1600-6143.2008.02272.x, PMID: 18510633

[61] Tavares MR Pavan IC Amaral CL Meneguello L Luchessi AD Simabuco FM. The S6K protein family in health and disease. Life Sci. (2015) 131:1–10. doi: 10.1016/j.lfs.2015.03.001, PMID: 25818187

[62] Cho HJ Park J Lee HW Lee YS Kim JB. Regulation of adipocyte differentiation and insulin action with rapamycin. Biochem Biophys Res Commun. (2004) 321:942–8. doi: 10.1016/j.bbrc.2004.07.050, PMID: 15358118

[63] Gagnon A Lau S Sorisky A. Rapamycin-sensitive phase of 3T3-L1 preadipocyte differentiation after clonal expansion. J Cell Physiol. (2001) 189:14–22. doi: 10.1002/jcp.1132, PMID: 11573200

[64] Yeh WC Bierer BE McKnight SL. Rapamycin inhibits clonal expansion and adipogenic differentiation of 3T3-L1 cells. Proc Natl Acad Sci U S A. (1995) 92:11086–90. PMID: 7479942 10.1073/pnas.92.24.11086 PMC40576

[65] Catalán V Gómez-Ambrosi J Rodríguez A Ramírez B Andrada P Rotellar F. Expression of S6K1 in human visceral adipose tissue is upregulated in obesity and related to insulin resistance and inflammation. Acta Diabetol. (2015) 52:257–66. doi: 10.1007/s00592-014-0632-9, PMID: 25118997

[66] Kim JE Chen J. Regulation of peroxisome proliferator-activated receptor-gamma activity by mammalian target of rapamycin and amino acids in adipogenesis. Diabetes. (2004) 53:2748–56. doi: 10.2337/diabetes.53.11.2748, PMID: 15504954

[67] Lastres-Becker I Nonis D Eich F Klinkenberg M Gorospe M Kötter P. Mammalian ataxin-2 modulates translation control at the pre-initiation complex via PI3K/mTOR and is induced by starvation. Biochim Biophys Acta. (2016) 1862:1558–69. doi: 10.1016/j.bbadis.2016.05.017, PMID: 27240544 PMC4967000

[68] Le Bacquer O Petroulakis E Paglialunga S Poulin F Richard D Cianflone K. Elevated sensitivity to diet-induced obesity and insulin resistance in mice lacking 4E-BP1 and 4E-BP2. J Clin Invest. (2007) 117:387–96. doi: 10.1172/JCI29528, PMID: 17273556 PMC1783830

[69] Kim K Pyo S Um SH. S6 kinase 2 deficiency enhances ketone body production and increases peroxisome proliferator-activated receptor alpha activity in the liver. Hepatology. (2012) 55:1727–37. doi: 10.1002/hep.25537, PMID: 22183976

[70] Um SH Frigerio F Watanabe M Picard F Joaquin M Sticker M. Absence of S6K1 protects against age- and diet-induced obesity while enhancing insulin sensitivity. Nature. (2004) 431:200–5. doi: 10.1038/nature02866 15306821

[71] Maechler P Wollheim CB. Mitochondrial function in normal and diabetic beta-cells. Nature. (2001) 414:807–12. doi: 10.1038/414807a 11742413

[72] Yuan T Lupse B Maedler K Ardestani A. mTORC2 signaling: a path for pancreatic β Cell's growth and function. J Mol Biol. (2018) 430:904–18. doi: 10.1016/j.jmb.2018.02.013, PMID: 29481838

[73] Kumar A Lawrence JC Jr Jung DY Ko HJ Keller SR Kim JK. Fat cell-specific ablation of rictor in mice impairs insulin-regulated fat cell and whole-body glucose and lipid metabolism. Diabetes. (2010) 59:1397–406. doi: 10.2337/db09-1061, PMID: 20332342 PMC2874700

[74] Fang Y Westbrook R Hill C Boparai RK Arum O Spong A. Duration of rapamycin treatment has differential effects on metabolism in mice. Cell Metab. (2013) 17:456–62. doi: 10.1016/j.cmet.2013.02.008, PMID: 23473038 PMC3658445

[75] Duijnhoven EMV Boots JMM Christiaans MHL Wolffenbuttel BHR Hooff JPV. Influence of tacrolimus on glucose metabolism before and after renal transplantation: a prospective study. J Am Soc Nephrol. (2001) 12:583–8. doi: 10.1681/ASN.V123583, PMID: 11181807

[76] Larsen JL Bennett RG Burkman T Ramirez AL Yamamoto S Gulizia J. Tacrolimus and sirolimus cause insulin resistance in normal Sprague dawley rats. Transplantation. (2006) 82:466–70. doi: 10.1097/01.tp.0000229384.22217.15, PMID: 16926589

[77] Oetjen E Baun D Beimesche S Krause D Cierny I Blume R. Inhibition of human insulin gene transcription by the immunosuppressive drugs cyclosporin a and tacrolimus in primary, mature islets of transgenic mice. Mol Pharmacol. (2003) 63:1289–95. doi: 10.1124/mol.63.6.1289, PMID: 12761338

[78] Radu RG Fujimoto S Mukai E Takehiro M Shimono D Nabe K. Tacrolimus suppresses glucose-induced insulin release from pancreatic islets by reducing glucokinase activity. Am J Physiol Endocrinol Metab. (2005) 288:E365–71. doi: 10.1152/ajpendo.00390.2004, PMID: 15479952

[79] Redmon JB Olson LK Armstrong MB Greene MJ Robertson RP. Effects of tacrolimus (FK506) on human insulin gene expression, insulin mRNA levels, and insulin secretion in HIT-T15 cells. J Clin Invest. (1996) 98:2786–93. doi: 10.1172/JCI119105, PMID: 8981925 PMC507744

[80] Chakkera HA Mandarino LJ. Calcineurin inhibition and new-onset diabetes mellitus after transplantation. Transplantation. (2013) 95:647–52. doi: 10.1097/TP.0b013e31826e592e, PMID: 23076551 PMC3884894

[81] Soleimanpour SA Crutchlow MF Ferrari AM Raum JC Groff DN Rankin MM. Calcineurin signaling regulates human islet {beta}-cell survival. J Biol Chem. (2010) 285:40050–9. doi: 10.1074/jbc.M110.154955, PMID: 20943662 PMC3000987

[82] Dai C Hang Y Shostak A Poffenberger G Hart N Prasad N. Age-dependent human β cell proliferation induced by glucagon-like peptide 1 and calcineurin signaling. J Clin Invest. (2017) 127:3835–44. doi: 10.1172/JCI91761, PMID: 28920919 PMC5617654

[83] Heit JJ Apelqvist AA Gu X Winslow MM Neilson JR Crabtree GR. Calcineurin/NFAT signalling regulates pancreatic beta-cell growth and function. Nature. (2006) 443:345–9. doi: 10.1038/nature05097, PMID: 16988714

[84] Johnson JD Ao Z Ao P Li H Dai LJ He Z. Different effects of FK506, rapamycin, and mycophenolate mofetil on glucose-stimulated insulin release and apoptosis in human islets. Cell Transplant. (2009) 18:833–45. doi: 10.3727/096368909X471198, PMID: 19500470

[85] Drachenberg CB Klassen DK Weir MR Wiland A Fink JC Bartlett ST. Islet cell damage associated with tacrolimus and cyclosporine: morphological features in pancreas allograft biopsies and clinical correlation. Transplantation. (1999) 68:396–402. doi: 10.1097/00007890-199908150-00012, PMID: 10459544

[86] Ajabnoor MA El-Naggar MM Elayat AA Abdulrafee A. Functional and morphological study of cultured pancreatic islets treated with cyclosporine. Life Sci. (2007) 80:345–55. doi: 10.1016/j.lfs.2006.09.034, PMID: 17074365

[87] Rostambeigi N Lanza IR Dzeja PP Deeds MC Irving BA Reddi HV. Unique cellular and mitochondrial defects mediate FK506-induced islet β-cell dysfunction. Transplantation. (2011) 91:615–23. doi: 10.1097/TP.0b013e3182094a33, PMID: 21200364 PMC3339767

[88] Vincenti F Friman S Scheuermann E Rostaing L Jenssen T Campistol JM. DIRECT (diabetes incidence after renal transplantation: Neoral C monitoring versus tacrolimus) Investigators. Results of an international, randomized trial comparing glucose metabolism disorders and outcome with cyclosporine versus tacrolimus. Am J Transplant. (2007) 7:1506–14. doi: 10.1111/j.1600-6143.2007.01749.x, PMID: 17359512

[89] Ciancio G Burke GW Gaynor JJ Ruiz P Roth D Kupin W. A randomized long-term trial of tacrolimus/sirolimus versus tacrolimums/mycophenolate versus cyclosporine/sirolimus in renal transplantation: three-year analysis. Transplantation. (2006) 81:845–52. doi: 10.1097/01.tp.0000203894.53714.27, PMID: 16570006

[90] Chakkera HA Kudva Y Kaplan B. Calcineurin inhibitors: pharmacologic mechanisms impacting both insulin resistance and insulin secretion leading to glucose dysregulation and diabetes mellitus. Clin Pharmacol Ther. (2017) 101:114–20. doi: 10.1002/cpt.546 27804122

[91] Ekberg H Tedesco-Silva H Demirbas A Vítko S Nashan B Gürkan A. ELITE-Symphony study. Reduced exposure to calcineurin inhibitors in renal transplantation. N Engl J Med. (2007) 357:2562–75. doi: 10.1056/NEJMoa067411 18094377

[92] Ekberg H Bernasconi C Tedesco-Silva H Vítko S Hugo C Demirbas A. Calcineurin inhibitor minimization in the Symphony study: observational results 3 years after transplantation. Am J Transplant. (2009) 9:1876–85. doi: 10.1111/j.1600-6143.2009.02726.x, PMID: 19563339

[93] Büchler M Caillard S Barbier S Thervet E Toupance O Mazouz H. Sirolimus versus cyclosporine in kidney recipients receiving thymoglobulin, mycophenolate mofetil and a 6-month course of steroids. Am J Transplant. (2007) 7:2522–31. doi: 10.1111/j.1600-6143.2007.01976.x 17868057

[94] Lebranchu Y Snanoudj R Toupance O Weestel PF Hurault de Ligny B Buchler M. Five-year results of a randomized trial comparing de novo sirolimus and cyclosporine in renal transplantation: the SPIESSER study. Am J Transplant. (2012) 12:1801–10. doi: 10.1111/j.1600-6143.2012.04036.x, PMID: 22486815

[95] Kreis H Cisterne JM Land W Wramner L Squifflet JP Abramowicz D. Sirolimus in association with mycophenolate mofetil induction for the prevention of acute graft rejection in renal allograft recipients. Transplantation. (2000) 69:1252–60. doi: 10.1097/00007890-200004150-00009, PMID: 10798738

[96] Flechner SM Glyda M Cockfield S Grinyó J Legendre C Russ G. The ORION study: comparison of two sirolimus-based regimens versus tacrolimus and mycophenolate mofetil in renal allograft recipients. Am J Transplant. (2011) 11:1633–44. doi: 10.1111/j.1600-6143.2011.03573.x, PMID: 21668635

[97] Teutonico A Schena PF Di Paolo S. Glucose metabolism in renal transplant recipients: effect of calcineurin inhibitor withdrawal and conversion to sirolimus. J Am Soc Nephrol. (2005) 16:3128–35. doi: 10.1681/ASN.2005050487 16107580

[98] Lebranchu Y Thierry A Toupance O Westeel PF Etienne I Thervet E. Efficacy on renal function of early conversion from cyclosporine to sirolimus 3 months after renal transplantation: concept study. Am J Transplant. (2009) 9:1115–23. doi: 10.1111/j.1600-6143.2009.02615.x, PMID: 19422337

[99] Budde K Becker T Arns W Sommerer C Reinke P Eisenberger U. Everolimus-based, calcineurin-inhibitor-free regimen in recipients of de-novo kidney transplants: an open-label, randomised, controlled trial. Lancet. (2011) 377:837–47. doi: 10.1016/S0140-6736(10)62318-5 21334736

[100] Schena FP Pascoe MD Alberu J del Carmen Rial M Oberbauer R Brennan DC. Conversion from calcineurin inhibitors to sirolimus maintenance therapy in renal allograft recipients: 24-month efficacy and safety results from the CONVERT trial. Transplantation. (2009) 87:233–42. doi: 10.1097/TP.0b013e3181927a41 19155978

[101] Holdaas H Rostaing L Serón D Cole E Chapman J Fellstrøm B. Conversion of long-term kidney transplant recipients from calcineurin inhibitor therapy to everolimus: a randomized, multicenter, 24-month study. Transplantation. (2011) 92:410–8. doi: 10.1097/TP.0b013e318224c12d 21697773

[102] Veroux M Tallarita T Corona D Sinagra N Giaquinta A Zerbo D. Conversion to sirolimus therapy in kidney transplant recipients with new onset diabetes mellitus after transplantation. Clin Dev Immunol. (2013) 2013:496974:1–7. doi: 10.1155/2013/496974 23762090 PMC3671526

[103] Vítko S Margreiter R Weimar W Dantal J Kuypers D Winkler M. Three-year efficacy and safety results from a study of everolimus versus mycophenolate mofetil in de novo renal transplant patients. Am J Transplant. (2005) 5:2521–30. doi: 10.1111/j.1600-6143.2005.01063.x 16162203

[104] Vitko S Wlodarczyk Z Kyllönen L Czajkowski Z Margreiter R Backman L. Tacrolimus combined with two different dosages of sirolimus in kidney transplantation: results of a multicenter study. Am J Transplant. (2006) 6:531–8. doi: 10.1111/j.1600-6143.2005.01193.x 16468962

[105] Van Gurp E Bustamante J Franco A Rostaing L Becker T Rondeau E. Comparable renal function at 6 months with tacrolimus combined with fixed-dose Sirolimus or MMF: results of a randomized multicenter trial in renal transplantation. J Transp Secur. (2010) 2010:731426:1–10. doi: 10.1155/2010/731426 PMC2952911 20953372

[106] Guerra G Ciancio G Gaynor JJ Zarak A Brown R Hanson L. Randomized trial of immunosuppressive regimens in renal transplantation. J Am Soc Nephrol. (2011) 22:1758–68. doi: 10.1681/ASN.2011010006, PMID: 21807891 PMC3171946

[107] Gallon L Perico N Dimitrov BD Winoto J Remuzzi G Leventhal J. Long-term renal allograft function on a tacrolimus-based, pred-free maintenance immunosuppression comparing sirolimus vs. MMF Am J Transplant. (2006) 6:1617–23. doi: 10.1111/j.1600-6143.2006.01340.x, PMID: 16827862

[108] Chhabra D Skaro AI Leventhal JR Dalal P Shah G Wang E. Long-term kidney allograft function and survival in prednisone-free regimens: tacrolimus/mycophenolate mofetil versus tacrolimus/sirolimus. Clin J Am Soc Nephrol. (2012) 7:504–12. doi: 10.2215/CJN.06940711, PMID: 22282478 PMC3302671

[109] Tedesco Silva H Jr CD Johnston T Lackova E Mange K Panis C. Everolimus plus reduced-exposure CsA versus mycophenolic acid plus standard-exposure CsA in renal-transplant recipients. Am J Transplant. (2010) 10:1401–13. doi: 10.1111/j.1600-6143.2010.03129.x, PMID: 20455882

[110] Berger SP Sommerer C Witzke O Tedesco H Chadban S Mulgaonkar S. Two-year outcomes in de novo renal transplant recipients receiving everolimus-facilitated calcineurin inhibitor reduction regimen from the TRANSFORM study. Am J Transplant. (2019) 19:3018–34. doi: 10.1111/ajt.15480, PMID: 31152476

[111] Murakami N Riella LV Funakoshi T. Risk of metabolic complications in kidney transplantation after conversion to mTOR inhibitor: a systematic review and meta-analysis. Am J Transplant. (2014) 14:2317–27. doi: 10.1111/ajt.12852, PMID: 25146383

[112] Howell JJ Hellberg K Turner M Talbott G Kolar MJ Ross DS. Metformin inhibits hepatic mTORC1 signaling via dose-dependent mechanisms involving AMPK and the TSC complex. Cell Metab. (2017) 25:463–71. doi: 10.1016/j.cmet.2016.12.009, PMID: 28089566 PMC5299044

[113] Kotha S Lawendy B Asim S Gomes C Yu J Orchanian-Cheff A. Impact of immunosuppression on incidence of post-transplant diabetes mellitus in solid organ transplant recipients: systematic review and meta-analysis. World J Transplant. (2021) 11:432–42. doi: 10.5500/wjt.v11.i10.432, PMID: 34722172 PMC8529944

